# Long-term results with Game-Based Physiotherapy (GBPT) according to Schroth in children with Juvenile Idiopathic Scoliosis (JIS)

**DOI:** 10.1186/1748-7161-8-S2-O46

**Published:** 2013-09-18

**Authors:** Petra Gröbl, Vinay Saraph

**Affiliations:** 1 Degree Program Physiotherapy, FH JOANNEUM Graz, Austria, LKH Univ.-Klinikum Graz, Austria

## Background

Apart from correcting inconvenient everyday postures, therapeutic exercises are the key to positive development of children with JIS.

## Purpose

The goal is to foster healing by offering motivating exercises combined with regular training aids. The available computer program provides valuable information on the exercise regime and can be used to monitor and evaluate treatment processes. Precise performance of exercises and exercise times are used more efficiently. Hypothesis: Patients who train with GBPT have, on average, a significantly better therapy outcome than those who are treated conventionally without a measuring instrument. 

## Methods

An intervention and control group consisted of 13 children with JIS between the ages of 9 and 13 years (Cobb curve of 23° +/- 4°, bracing with Cheneau braces). Both groups exercised for six months according to the “Schroth” concept; two standardized exercises were chosen for the study group and guided by the computer program GBPT, so that minor movements of feet could be detected in a narrow range, but not for the control group. 

## Results

Comparing children of the intervention group with those of the control group revealed a difference in the Cobb angle after the observed period of six months exercising. The comparison of the two test groups showed a significant difference in the lumbar spine results of 3.5° (p=0.049). The evaluation showed a clear tendency of the test group in angle improvement, whereas the control group showed a deterioration of the Cobb angle during the test phase. 

## Conclusions and discussion

The results of the long-term study led to the conclusion that the exercise, “Muscle Cylinder in Side Position” of the Schroth concept, supported by the game track, seemed to have a positive influence on the Cobb curve of the spine in the treatment of children with JIS. 
